# Screening for GmRCD1-Interacting Proteins in Glycine Max and Characterization of the GmRCD1-GmNAC058 Interaction

**DOI:** 10.3390/ijms26167760

**Published:** 2025-08-11

**Authors:** Yupeng Li, Youda Bu, Yun Liu, Guobao Liu

**Affiliations:** 1 Guangdong Provincial Key Laboratory for Plant Epigenetics, College of Life Sciences and Oceanography, Shenzhen University, Shenzhen 518060, China; 2300251031@email.szu.edu.cn (Y.L.); buyouda@catas.cn (Y.B.);; 2 Haikou Experimental Station Chinese Academy of Tropical Agricultural Sciences, Haikou 571101, China

**Keywords:** GmRCD1, GmNAC058, yeast two-hybrid

## Abstract

In response to abiotic stress, plants utilize hub protein-mediated signaling networks, with members of the SIMILAR TO RCD ONE (SRO) protein family playing a pivotal role in regulating stress resistance pathways. This study investigates the functional role of the soybean GmRCD1 protein and its interaction mechanisms to elucidate its molecular regulatory network in stress resistance responses. By employing yeast two-hybrid technology to screen a soybean cDNA library under high-salt stress conditions, 17 potential interacting proteins were identified, which include NAC transcription factors (e.g., GmNAC058), ubiquitin–proteasome proteins, and ribosomal proteins. Subsequent validation using GST pull-down and bimolecular fluorescence complementation assays confirmed the direct interaction between GmRCD1 and GmNAC058, which is mediated by the RST domain of GmRCD1 and the C-terminal disordered region (amino acids 288–323) of GmNAC058. Subcellular localization studies revealed that both proteins are nuclear-localized, aligning with their roles in transcriptional regulation. Furthermore, PAR binding assays demonstrated that both GmRCD1 and AtRCD1 can bind to PAR polymers; however, PARP activity analysis revealed that neither protein exhibits catalytic activity, indicating their participation in stress responses via non-enzymatic mechanisms. This study represents the first to elucidate the interaction network and structural basis between soybean GmRCD1 and GmNAC058, providing crucial theoretical support for understanding the multifunctional roles of plant hub proteins in stress resistance regulation and for molecular breeding in soybean.

## 1. Introduction

Abiotic stresses, such as salinity, drought, and low temperatures, frequently impact agricultural development, posing significant threats to crop growth and yield [1]. In response to stress, plants initiate metabolic changes and activate stress-related gene expression to protect themselves, a process involving receptor proteins, signaling proteins, transcription factors, and effector proteins [2]. Transcription factors are particularly crucial as making them a focal point in plant stress resistance research [3,4]. During stress responses, hub proteins facilitate efficient intracellular communication by regulating downstream transcription factors, thereby enhancing the plant’s ability to adapt to environmental changes [5,6].

The SRO protein family members are widely involved in the normal growth and development of plants and also play a crucial role in the plant’s response to drought, salt, and heavy metals [7]. The SRO protein family in Arabidopsis comprises six members, including AtRCD1 and AtSRO1-5 [8]. AtRCD1 is identified as a novel hub protein due to its extensive interactions with various transcription factors, while AtSRO1 is involved in the response to osmotic and oxidative stress [9]. The SRO protein family members exhibit structural similarity, with RCD1 and SRO1 showing the highest homology at more than 65.0% [10]. Plant SRO family members are highly conserved structurally; notably, only RCD1 and SRO1 feature the WWE domain at the N-terminus, whereas all members possess the catalytic core PARP (poly ADP-ribose polymerase) domain and the C-terminal RST (RCD-SRO-TAF4) domain [11]. The WWE domain is predicted to mediate protein–protein interactions. Although WWE domain-containing proteins are prevalent in eukaryotes, the SRO protein family is the only known plant protein family that incorporates the WWE domain [12]. The catalytic core PARP domain, which is the most conserved domain within the SRO family, is associated with DNA repair functions in most eukaryotes [13]. The RST domain is likely responsible for mediating interactions with transcription factors, and this interaction occurs independently of other domains [14].

The initial study on the hub protein RCD1 demonstrated its capacity to enhance yeast’s tolerance to damage induced by reactive oxygen species (ROS) [15]. The Arabidopsis AtRCD1 gene mutant *rcd1-1* exhibits phenotypes such as dwarfism, early bolting, and leaf deformity [16], while the *rcd1-3* mutant shows severe developmental impairments, including disrupted cell differentiation [17], underscoring the critical role of RCD1 in plant growth and development. Overexpression of AtRCD1 in Arabidopsis resulted in phenotypes intermediate between those of the wild type and the mutant, supporting the notion that appropriate expression levels of RCD1 are essential for normal plant development [18]. Under normal conditions, the RCD1 protein is localized in the nucleus; however, it can translocate to the cytoplasm in response to salt or ROS stress [19], suggesting that RCD1 may contribute to stress responses by changing its subcellular localization under stress conditions. The *rcd1-1* mutant displayed significantly enhanced resistance to methyl viologen (MV) stress but was hypersensitive to salt and hydrogen peroxide [20]. Additionally, the *rcd1-2* mutant exhibited a fourfold increase in resistance to methyl viologen, a non-selective contact herbicide. Under ultraviolet stress, the *rcd1-2* mutant showed significantly reduced levels of DNA cyclobutane pyrimidine dimers compared to the wild type, indicating enhanced DNA damage repair capabilities [20,21]. Furthermore, RCD1 directly interacts with the Na^+^/H^+^ antiporter SOS1, negatively regulating the response of Arabidopsis to sodium ion toxicity [19]. Moreover, RCD1 may negatively regulate plant responses to cadmium stress and exposure to mercury chloride (HgCl_2_) [21].

Transcription factors ANAC013 and ANAC017 regulate the expression of mitochondrial dysfunction stimulon (MDS) proteins, which protect mitochondria and chloroplasts from oxidative stress-induced damage [22,23]. Recent studies have shown that Arabidopsis RCD1 interacts with transcription factors ANAC013 and ANAC017, thereby suppressing their activity and negatively regulating the plant’s response to oxidative stress [24,25]. The transcription factor DREB2A is a critical regulator of drought and heat tolerance in plants, and its function depends on the presence of specific structural domains [26]. AtRCD1 interacts with the DREB2A transcription factor within the 136-165 amino acid region. Under conditions of drought and heat stress, plants generate a splice variant of DREB2A that lacks the domain required for interaction with RCD1. This splice variant accumulates during heat stress, whereas RCD1 is rapidly degraded under these conditions, indicating that RCD1 typically functions as a negative regulator of DREB2A [20,27].

In summary, the hub protein RCD1 is involved in regulating multiple abiotic stress responses in plants through a complex protein network, rather than being limited to a single stress resistance pathway. Loss of AtRCD1 function affects the expression of over 500 genes [5], including transcription factor families such as NAC, DREB, MYB, and HLH [28], most of which are associated with plant stress resistance. This highlights the essential role of RCD1 in regulating diverse signaling pathways.

Although substantial progress has been made in elucidating the role of RCD1 in model organisms such as Arabidopsis and rice, its physiological function in soybean remains largely unexplored. Furthermore, the roles of RCD1′s conserved domains—PARP, RST, and WWE—in plant stress resistance remain controversial. In this study, we employed the yeast two-hybrid system to identify potential interacting partners of GmRCD1 and examined how these interactions, mediated by conserved domains, affect transcription factors. The findings aim to establish a theoretical basis for developing innovative strategies to improve plant stress resistance.

## 2. Results

### 2.1. Genome-Wide Identification and Bioinformatics Analysis of the Soybean SRO Family

**Figure 1:**
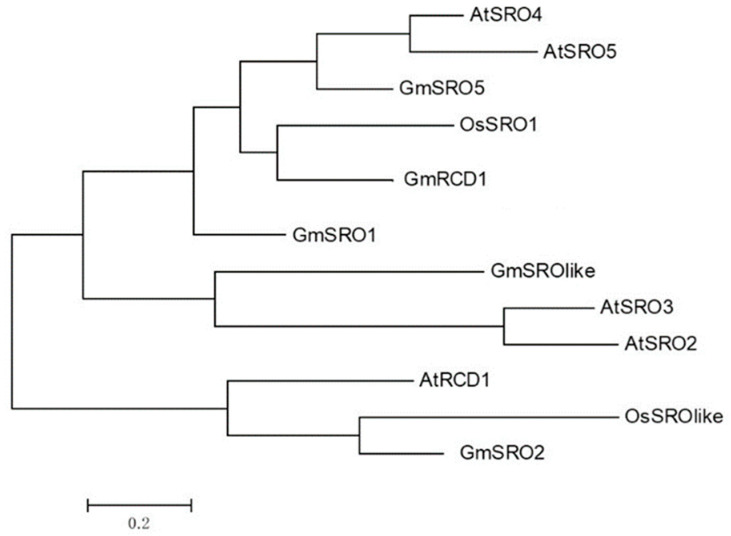
Phylogenetic analysis of soybean GmSRO family and other plant SRO families. Using the Dayhoff model in MEGA6.06 software to calculate evolutionary distances, a phylogenetic tree with 1000 bootstrap replicates was constructed. The genetic distance between GmRCD1 and OsSRO1 is relatively small.

The Pfam database (http://pfam.xfam.org/, accessed on 19 March 2019) was utilized to identify soybean sequences containing both the PARP (PF00644) and RST (PF12174) domains (*p* value = 0.001), with candidate sequences further verified using the NCBI database (https://www.ncbi.nlm.nih.gov/, accessed on 19 March 2019). This analysis yielded five non-redundant soybean sequences: GmRCD1, GmSRO1, GmSRO2, GmSRO5, and GmSRO-like. To explore the evolutionary relationships of the hub protein GmRCD1, a phylogenetic tree was constructed using SRO protein family members from soybean, Arabidopsis, and rice. Six SRO protein members were identified in Arabidopsis based on the conserved PARP-RST domain: AtRCD1 (At1g32230), AtSRO1 (At2g35510), AtSRO2 (At1g23550), AtSRO3 (At1g70440), AtSRO4 (At3g47720), and AtSRO5 (At5g62520). Additionally, two SRO proteins were identified in rice: OsSRO1 (Os03g0230300) and OsSRO-like (Os10g0577800). Evolutionary distances were calculated using the Dayhoff model in MEGA6.06 software, and a phylogenetic tree was constructed with 1000 bootstrap replicates to assess statistical support. The phylogenetic analysis revealed a small genetic distance between GmRCD1 and OsSRO1 (Figure 1), suggesting a close evolutionary relationship. Previous studies have shown that OsSRO1 interacts with multiple transcription factors and plays a crucial role in rice stress response. The ossro1-1 mutant, which lacks functional OsSRO1, exhibits a reduced response to chloroplast oxidative stress, indicating that GmRCD1 may similarly regulate stress resistance in soybean.

The protein sequence of GmRCD1, consisting of 583 amino acids, was retrieved from the NCBI database. Domain analysis using Pfam and NCBI databases confirmed that GmRCD1 contains three conserved domains: a WWE domain (amino acids 70–160), a PARP domain (amino acids 260–460), and an RST domain (amino acids 491–583).

### 2.2. Screening of GmRCD1-Interacting Proteins

**Figure 2:**
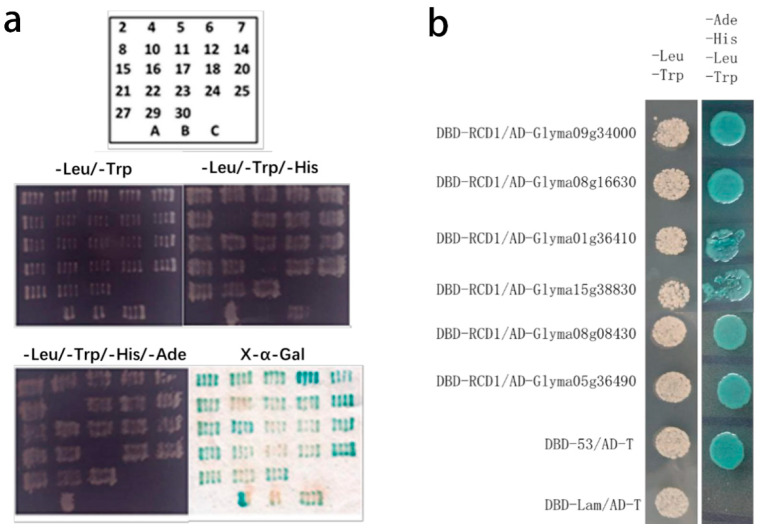
Confirmation of yeast two-hybrid interactions by retransformation. (**a**) Retransformation of 23 positive clones with controls A: pGBKT7-53 + pGADT7-T, positive control B: pGBKT7-Lam + pGADT7-T, negative control C: pGBKT7-GmRCD1 + pGADT7, autoactivation control (**b**) Verification of interactions using full-length constructs of six positive clones.

A mixed root and leaf cDNA library was previously constructed from 15-day-old soybean seedlings exposed to high salt stress (150 mM NaCl). This specific stress concentration triggers protective biochemical and molecular responses in plants, rendering the system amenable to molecular investigation [29,30]. Co-transformation of the pGBKT7-GmRCD1 bait vector and the soybean cDNA library into Y2H GOLD yeast cells, followed by validation through fragment reversion, resulted in 23 positive clones (Figure 2a).

The 23 positive clones were sequenced, and their sequences were subjected to BLAST (https://blast.ncbi.nlm.nih.gov/Blast.cgi, accessed on 19 November 2018) analysis against the NCBI database to identify corresponding full-length gene sequences. Sequences with redundant genes or incorrect open reading frames were excluded from further analysis. Comparative analysis identified 17 potential interacting partners, which were categorized based on their biological functions (Table 1).

Six candidate genes (Glyma09g34000, Glyma08g16630, Glyma01g36410, Glyma15g39830, Glyma08g08430, and Glyma05g36490) were cloned into the pGADT7 vector and verified via full-length reversion. These recombinant pGADT7 vectors were co-transformed with pGBKT7-GmRCD1 into Y2H GOLD yeast cells, resulting in six positive interactions (Figure 2b). Among the 17 candidate interacting proteins, Glyma04g39140 (GmNAC020) and Glyma08g16630 (GmNAC058) were identified as homologs of ANAC082 through sequence alignment. The homologous gene in Arabidopsis, ANAC082, is a key component of the stress-responsive ribosomal signaling pathway [31,32]. Consequently, GmNAC058 was selected for further investigation.

### 2.3. Verification of Interaction Between GmRCD1 and GmNAC058

**Figure 3:**
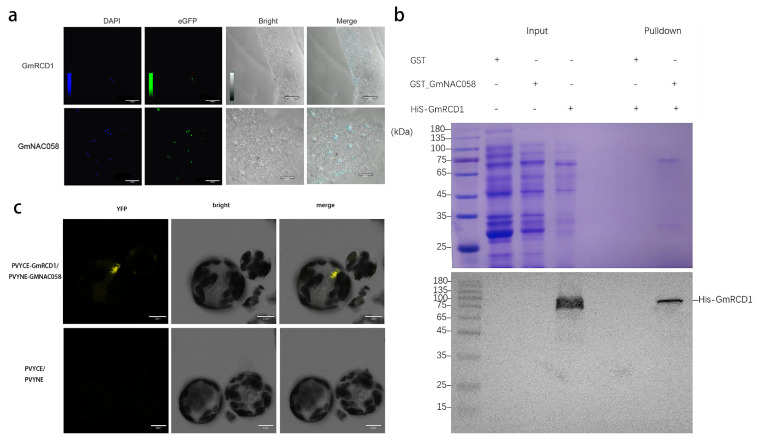
Verification of the interaction between GmRCD1 and GmNAC058 (**a**) Subcellular localization. DAPI staining revealed that the green fluorescence from eGFP co-localized with the blue fluorescence from DAPI in the nucleus, confirming that both GmRCD1 and GmNAC058 are predominantly located in the nucleus. (**b**) GST pull-down assay. The GST pull-down assay demonstrates the interaction between GmRCD1 and GmNAC058. Samples were resolved by SDS-PAGE, visualized by Coomassie blue staining (top), and analyzed by Western blotting using an anti-His antibody (bottom). (**c**) Bimolecular fluorescence complementation (BiFC) assay. The BiFC assay confirms the interaction between GmRCD1 and GmNAC058. Recombinant plasmids were introduced into Arabidopsis protoplasts, and YFP fluorescence was observed in the transformed protoplasts. The image is representative of three independent experiments.

The GmRCD1 and GmNAC058 genes were each cloned into the pCAMBIA2300-eGFP vector, and the subcellular localization of the GmRCD1-eGFP and GmNAC058-eGFP fusion proteins was assessed after transfection into tobacco. DAPI staining demonstrated co-localization of the green fluorescence from eGFP with the blue fluorescence from DAPI within the nucleus, thereby confirming the predominant nuclear localization of both GmRCD1 and GmNAC058. This localization pattern is consistent with the potential interaction between the two proteins, as illustrated in Figure 3a.

To verify the interaction between GmRCD1 and GmNAC058, the GmNAC058 gene was cloned into the pGEX4T-1 vector, while the GmRCD1 gene was cloned into the pET28a vector. After expression and purification, the recombinant proteins GST-GmNAC058 and His-GmRCD1 were successfully obtained. These recombinant proteins were then used in a GST pull-down assay, and the outcomes are shown in Figure 3b. Analysis by SDS-PAGE revealed that the sizes of GmNAC058 and GmRCD1 are very similar. Western blot analysis detected His-GmRCD1 in the sample containing GST-GmNAC058 but not in the GST control, thereby confirming a direct interaction between GmRCD1 and GmNAC058.

The interaction between GmRCD1 and GmNAC058 was further confirmed using bimolecular fluorescence complementation (BiFC) analysis. To further validate this interaction, Arabidopsis protoplasts were co-transformed with constructs encoding GmNAC058 fused to the N-terminal half of YFP (GmNAC058-PVYNE) and GmRCD1 fused to the C-terminal half of YFP (GmRCD1-PVYCE), and the presence of YFP fluorescence was observed. As shown in Figure 3c, YFP fluorescence was observed in Arabidopsis protoplasts co-expressing GmRCD1-PVYCE and GmNAC058-PVYNE, while no fluorescence was detected in the negative control (PVYCE and PVYNE alone), thus confirming the interaction between GmRCD1 and GmNAC058 within plant cells.

### 2.4. Identification of Interaction Domains Between GmRCD1 and GmNAC058

**Figure 4:**
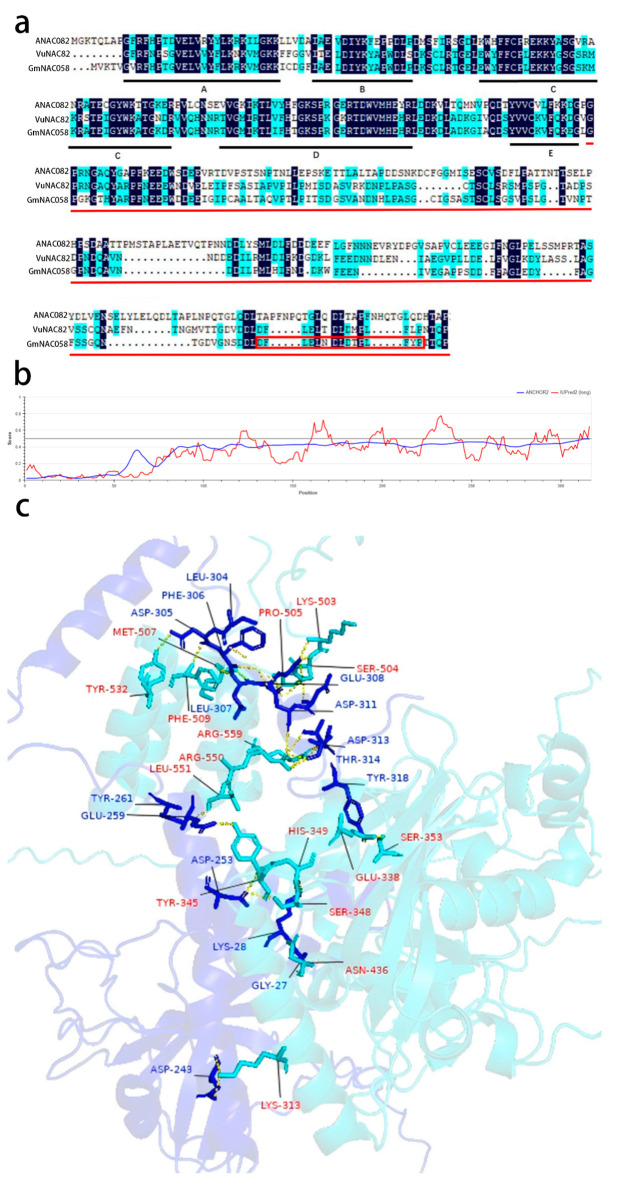
Identification of interaction domains between GmRCD1 and GmNAC058. (**a**) Sequence alignment of NAC82 from Arabidopsis, cowpea, and soybean. Black shading indicates highly conserved residues; blue shading indicates similar residues; black bold lines denote the A–E subdomains of the NAC domain; red lines highlight the GmNAC058 fragment interacting with GmRCD1 as identified by screening; the red box outlines a potential interaction domain. (**b**) Disorder analysis of GmNAC058. The graph indicates the predicted disordered sequence profile in red and the predicted disordered binding sites in blue. Predicted disordered binding sites are positions with a score higher than 0.5. The disordered structure of GmNAC058 is mainly concentrated at the C-terminus. (**c**) AlphaFold3-predicted (https://alphafoldserver.com/, accessed on 19 March 2025) protein interaction sites. The blue protein is GmNAC058, and the cyan protein is GmRCD1. The main binding site of GmRCD1 is located in the RST structural domain. There may also be some loose binding at other amino acid sites.

Sequence homology analysis of the transcription factor GmNAC058 revealed a high degree of similarity with proteins encoded by NAC82 in species including Arabidopsis, soybean, and cowpea. Amino acid sequence alignment using DNAMAN software (v5.2.9) demonstrated that the N-terminal region of GmNAC058, consisting of approximately 150 amino acids and including subdomains A through E, is highly conserved, a feature characteristic of typical NAC transcription factors. The red lines highlight the GmNAC058 fragment interacting with GmRCD1 as identified by screening (Figure 4a). The intrinsic disorder and potential binding sites for interacting proteins in GmNAC058 were analyzed using the online tool IUPred2A (https://iupred2a.elte.hu/, accessed on 18 March 2025). The analysis revealed that the C-terminal region of GmNAC058 is primarily disordered (Figure 4b). The region highlighted by the red box denotes a putative interaction binding site (Figure 4a).

**Figure 5:**
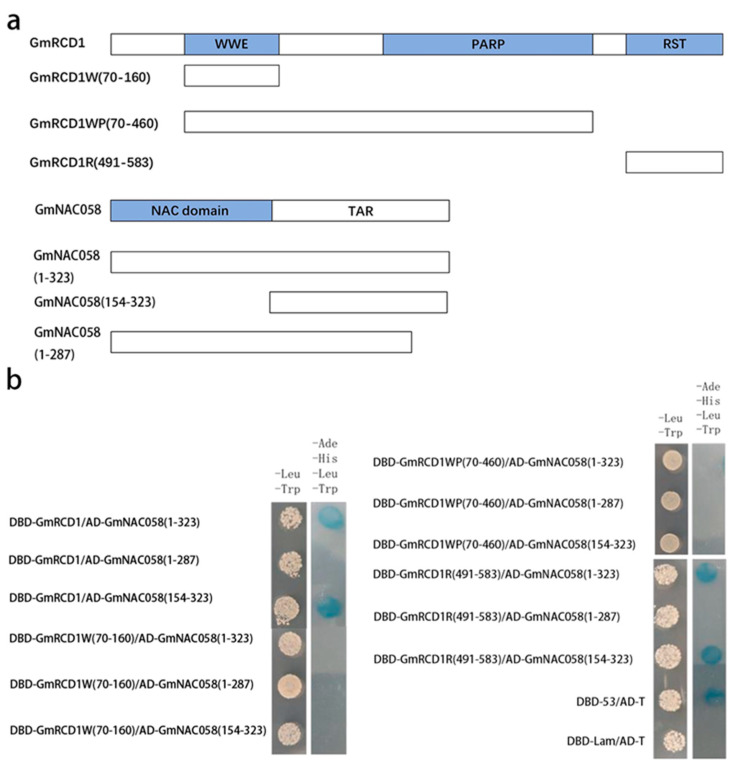
Mapping interaction domains of GmRCD1 and GmNAC058 using yeast two-hybrid. (**a**) Schematic representation of GmRCD1 and GmNAC058 Fragments. (**b**) Yeast growth assays with various fragment combinations. Only the combinations involving the full-length GmRCD1 or its RST domain with either the full-length GmNAC058 or its 154-323 fragment resulted in growth, whereas all other combinations did not.

The potential binding sites for the interaction between GmRCD1 and GmNAC058 were further validated using AlphaFold3, with the results presented in Figure 4c. The prediction indicates that GmRCD1 interacts with the disordered C-terminal tail of GmNAC058, primarily through its RST domain. Based on these findings, fragments GmRCD1W (residues 70–160), GmRCD1WP (residues 70–460), and GmRCD1R (residues 491–583) were cloned into the pGBKT7 vector, while GmNAC058 fragments (residues 154–323 and 1–287) were cloned into the pGADT7 vector. Various combinations of pGADT7-GmNAC058 and pGBKT7-GmRCD1 vectors, each harboring distinct fragments, were co-transformed into Y2H GOLD yeast cells and plated on the defective medium. The results (Figure 5) presented demonstrate that only combinations involving full-length GmRCD1 or its RST domain with either full-length GmNAC058 or its 154–323 fragment supported yeast growth, whereas all other combinations failed to do so. These observations suggest that the absence of the 288–323 segment in GmNAC058 impairs its interaction with GmRCD1. Consequently, it is confirmed that GmNAC058 interacts with the RST domain of GmRCD1 through its 288–323 motif.

### 2.5. PARP Activity Assay of GmRCD1

**Figure 6:**
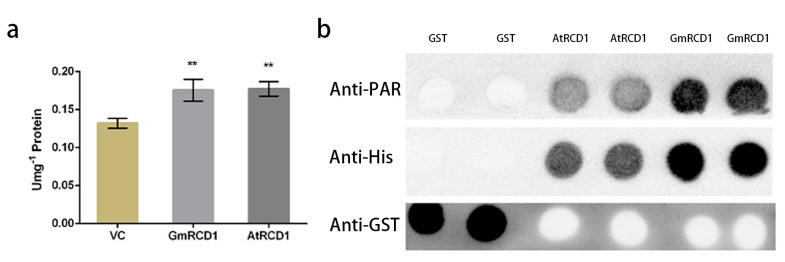
Functional analysis of the PARP domain. (**a**) PARP activity assay. GmRCD1′s PARP activity is comparable to AtRCD1′s and marginally above the pColdI total protein control (**b**) PAR binding assay. AtRCD1 and GmRCD1 proteins can bind to PAR, whereas GST proteins do not show binding to PAR. Mean ±SE are presented. Asterisks indicate values significantly different from the negative control, ** *p* value < 0.01, Student’s *t*-test.

The AtRCD1 protein possesses a PARP domain; however, research indicates that it does not exhibit PARP activity. To determine whether RCD1 proteins from various species share similar characteristics, we further examined the PARP activity of the GmRCD1 protein. The purified GmRCD1 protein was assayed for PARP activity, using total protein from pColdI and recombinant AtRCD1 protein as controls. As illustrated in Figure 6a, the PARP activity of GmRCD1 is similar to that of AtRCD1 and only slightly higher than the pColdI total protein control, indicating that neither the recombinant AtRCD1 nor GmRCD1 proteins exhibits PARP activity. Although plant RCD1 proteins contain a PARP domain, they do not employ PARP activity in their regulatory roles.

### 2.6. Assessment of PAR Binding Capacity in RCD1 Protein

Given that AtRCD1 and GmRCD1 proteins have been confirmed to lack PARP activity, a PAR dot blot assay was performed to further investigate their potential PAR binding capabilities. In vitro-expressed AtRCD1, GmRCD1, and GST proteins were utilized, with AtRCD1 serving as a positive control and GST as a negative control, to evaluate their PAR binding abilities. Anti-His and anti-GST antibodies were employed to confirm the presence and membrane binding of the recombinant proteins. Figure 6b shows that AtRCD1 binds to PAR, consistent with prior research, while GST does not exhibit PAR binding. Similarly, GmRCD1 also binds to PAR, indicating that both AtRCD1 and GmRCD1 possess the ability to bind PAR in vitro.

## 3. Discussion

### 3.1. Functional Investigation of GmRCD1-Interacting Proteins

The hub protein RCD1, recognized as a transcriptional co-regulator, interacts with various components to perform its functions [25]. For example, RCD1 acts as a critical node in the ROS-dependent signaling pathway that contributes to plant stress resistance [33]. In Arabidopsis, RCD1 regulates ROS-induced apoptosis through the modulation of transcription factors WRKY70 and SGT1b [34]. Additionally, RCD1 directly interacts with SOS1 to participate in the SOS signaling pathway [19], which is essential for maintaining sodium ion homeostasis under salt stress and represents a key mechanism for plant stress tolerance [35]. The phylogenetic analysis presented in Figure 1, which includes SRO protein family members from soybean, Arabidopsis, and rice, indicates that the homology between GmRCD1 and AtRCD1 is not particularly high. To demonstrate that GmRCD1 shares similar functions with AtRCD1, we expressed GmRCD1 in the Arabidopsis mutant *rcd1-3.* Under normal growth conditions, the *rcd1-3* mutant exhibits impaired development compared to wild-type Arabidopsis, characterized by stunted growth, curled leaves, and premature flowering. Overexpression of GmRCD1 in the *rcd1-3* mutant background complemented the mutant phenotype, resulting in plants that phenotypically resembled wild-type Arabidopsis (Appendix A). RCD1, a hub protein and key transcriptional co-regulator, is essential for plant stress resistance signaling and developmental regulation; it modulates these processes through interactions with various components, and our findings suggest that GmRCD1 possesses analogous functions to AtRCD1. Therefore, exploring genes that interact with RCD1 and their functions in plant stress resistance offers considerable potential for enhancing soybean molecular breeding.

In this study, we employed the yeast two-hybrid system to screen a soybean salt stress cDNA library for proteins that interact with GmRCD1. In addition to the primary focus on GmNAC058, several other potential interacting proteins were also identified.

In Arabidopsis, RCD1 typically exists as a stable monomer; however, under chloroplast ROS stress, it rapidly forms high-molecular-weight aggregates, and its abundance decreases. Further research demonstrates that cysteine residues in RCD1 regulate its oligomerization and stability in vivo [25,36]. Among the interacting partners of GmRCD1, GmSRO1, a homolog of GmRCD1, was identified through screening. Both GmRCD1 and GmSRO1 possess bipartite nuclear localization signals, while GmRCD1 also contains a putative nuclear export signal. Furthermore, BLAST sequence analysis using the NCBI database revealed that GmRCD1 contains multiple conserved cysteine residues, analogous to those in the plant resistance protein NONEXPRESSER OF PR GENES 1 (NPR1). The NPR1 protein forms dimers via disulfide bonds between conserved cysteine residues, regulating defense gene expression through dimer formation and dissociation [37]. Consequently, it is hypothesized that GmRCD1 exhibits a functional mechanism similar to NPR1, responding to abiotic stresses through homo- or heterodimer formation.

The ubiquitin–proteasome pathway is the principal mechanism for protein degradation in cells and is involved in virtually all biological processes, such as reproduction, gene regulation, tissue repair, and immune responses. In this pathway, proteins destined for degradation are sequentially modified by E1, E2, and E3 enzymes in conjunction with ubiquitin, culminating in their degradation by the proteasome [38]. In this study, we employed the yeast two-hybrid system to screen a soybean salt stress cDNA library for proteins that interact with GmRCD1. In addition to the primary focus on GmNAC058, several other potential interacting proteins were also identified, including proteins like Rpn13-like, DSK2, RING-type E3, and SINA-like 10, all of which are essential components of the ubiquitin–proteasome pathway. Rpn13 is a subunit of the 26S proteasome responsible for ubiquitin binding [39]. RING-type E3 ligases transfer ubiquitin, activated by E1 and E2 enzymes, to specific substrates and are the primary regulators of protein ubiquitination [40]. Current research shows that Arabidopsis has more than 1400 genes encoding E3 ubiquitin ligases, of which approximately 36% are RING-type E3 ligases. RING-type E3 ligases play a crucial role in plant responses to abiotic stress [41]. A study by Du et al. demonstrates that the soybean RING-type E3 ligase GmRFP1 decreases plant tolerance to cold stress by downregulating key proteins involved in the cold stress signaling pathway [42]. The rice protein OsDSK2a binds to polyubiquitin chains and interacts with the gibberellin (GA)-inactivating enzyme ELONGATED UPPERMOST INTERNODE (EUI), thereby facilitating the degradation of EUI through the ubiquitin–proteasome pathway. Under salt stress conditions, a decrease in OsDSK2a protein levels results in reduced EUI degradation, leading to EUI accumulation, lower GA levels, and consequently, inhibited plant growth [43]. GmRCD1 may interact with key components of the ubiquitin–proteasome system, thereby influencing protein degradation processes and contributing to the modulation of plant stress tolerance.

In addition to SRO1 and ubiquitin–proteasome system proteins, GmRCD1 interacts with the SQUAMOSA PROMOTER BINDING PROTEIN-LIKE 1 (SPL1) transcription factor, zinc finger domain-containing proteins, and NmrA domain-containing proteins, among others. Research indicates that the SPL1 transcription factor primarily regulates heat resistance during plant reproductive phases, working with SPL12 to enhance inflorescence heat tolerance under heat stress [44]. C_2_H_2_-type zinc finger proteins, essential transcription factors, regulate target gene expression by binding DNA or RNA, thus influencing plant growth, development, and stress responses; for example, ZFP245 enhances drought tolerance in rice by upregulating stress-responsive genes via ABA-dependent pathways [45]. NmrA domain-containing proteins enhance plant resilience to environmental stresses by regulating stress-responsive gene expression and physiological processes; notably, transgenic soybeans overexpressing GmNmrA6 exhibit elevated stress-related gene expression and greater tolerance to saline and oxidative conditions, possibly acting as negative transcriptional regulators in nitrogen metabolism [46]. GmRCD1 likely functions as a central component in soybean’s transcriptional regulatory network, coordinating gene expression through interactions with diverse transcription factors to mediate adaptive responses to environmental stresses.

These findings suggest that the hub protein RCD1 facilitates signal transduction to support plants during stress and senescence, potentially regulating multiple signaling pathways [47]. Therefore, examining how GmRCD1 interacts with these components to modulate plant stress resistance holds significant research and practical value, with ongoing studies underway.

### 3.2. Functional Characterization of Protein Domains

The NAC transcription factors constitute a group of plant-specific transcription factors. Their family members have been extensively investigated and are implicated in various biological processes, including plant growth and development, biotic defense, abiotic stress responses, and cell morphogenesis [48]. AtRCD1 interacts with ANAC transcription factors to modulate the functions of chloroplasts and mitochondria. Research demonstrates that SNAC1 regulates ROS levels by modulating OsSRO1c [49]. Recent investigations have established that ANAC082 in Arabidopsis contributes to the ribosomal stress response, with its signaling pathway exhibiting substantial overlap and similarity to the p53-MDM2 pathway activated in animal ribosomal stress responses [50]. Our study is the first to demonstrate that soybean GmRCD1 interacts with GmNAC058, a finding substantiated by multiple experimental approaches. Coincidentally, the interaction between the proteins AtRCD1 and ANAC082 in Arabidopsis was identified using the yeast two-hybrid [17]; however, further research on this interaction has been limited. These findings indicate that the interaction between RCD1 and NAC82 may be conserved throughout the plant kingdom. This interaction likely contributes to the plant’s ribosomal stress signaling pathway, providing valuable insights into the molecular mechanisms and physiological importance of plant ribosomal stress responses.

Intrinsically disordered proteins, despite lacking a stable three-dimensional structure, play a crucial role in protein functionality. The disordered regions in these proteins are essential for their function, especially in transcription factors, where they can remain disordered or adopt specific conformations to interact with other proteins and regulate transcription [51]. In barley, the transcription factor HvNAC013 possesses a disordered C-terminal region containing four conserved motifs: LP, DV, YF, and RR. The LP motif is responsible for transcriptional activation, whereas the YF-RR region forms an α-helix to interact with Hv-RCD1, highlighting the functional significance of disordered domains [52]. This characteristic elucidates how hub proteins like RCD1 can interact with multiple NAC transcription factors. Yeast two-hybrid assays confirmed that Arabidopsis RCD1 interacts with several plant stress-related proteins via its RST domain, including the transcription factors ANAC017 and ANAC013, as well as the Na^+^/H^+^ antiporter SOS1, underscoring its versatility in protein interactions [19,24,25]. Helena et al. determined the ^1^H, ^13^C, and ^15^N chemical shifts of the RCD1 protein’s RST domain and its complex with DREB2A, with the data deposited in the BioMagResBank (https://bmrb.io/, accessed on 14 May 2019) under accession number 27034 [53]. Furthermore, recent studies have revealed that the conserved RST domain of RCD1 can bind to disordered proteins or various structural polypeptides, including α-helices, through three anchor points within its TAF4-TAFH subdomain [54]. This study demonstrated, via yeast two-hybrid experiments, that GmNAC058 interacts with the conserved RST domain of GmRCD1 through its C-terminal motif spanning residues 288-323. Either the standalone 288-323 motif or the conserved RST domain is sufficient to mediate protein interactions. This discovery provides a molecular foundation for understanding how soybean NAC transcription factors, particularly those with intrinsically disordered regions, regulate gene transcription, thereby enhancing our theoretical understanding of transcription factors in modulating plant responses to abiotic stress.

PARylation serves as a crucial platform for various post-translational modifications, including ubiquitination. PARP enzymes can recruit the E3 ligase RFN146, thereby facilitating PARylation-induced ubiquitination [55]. In plant proteins, the WWE domain is recognized as a binding region for PARP [56]. Despite the existence of a functional coding system based on ADP-ribosylation, which involves the enzymatic addition and removal of ADP-ribose units by PARP and PARG, the absence of ‘reader’ proteins with ADP-ribose binding domains in plants has hindered the complete establishment of an ADP-ribosylation-based functional coding system [57]. SRO1 family proteins possess WWE domains that can bind iso-ADP; however, due to mutations in the catalytic triad, the SRO family lacks ADP-ribosylation activity and is thus considered to be devoid of PARP activity. There has been controversy regarding whether the wheat SRO protein Ta-sro1 exhibits PARP activity. Liu et al. initially reported that Ta-sro1 possesses PARP activity, as detected in vitro using a commercial kit, and associated this activity with enhanced salt tolerance and seedling growth in wheat through mutant and overexpression studies [58]. However, Vogt et al. conclusively showed, through thermal stability and ^32^P-NAD^+^ binding assays, that Ta-sro1 is unable to bind NAD^+^, and neither auto-ADP-ribosylation assays nor detection with the Trevigen kit revealed any PARP activity. Additionally, heterologous expression of Ta-sro1 in tobacco did not produce ADP-ribosylation signals, further confirming the absence of typical PARP activity [59]. Both Liu and Vogt confirmed that the SR3 wheat line exhibits elevated ROS levels and enhanced salt tolerance; however, Vogt posits that the salt tolerance and growth-promoting effects of Ta-sro1 are more likely mediated through the regulation of ROS or transcription factors rather than through PARP activity [58,59]. Therefore, it is hypothesized that the PARP-like domain in the plant SRO family may have evolved from PARP but has lost its typical catalytic function due to functional divergence, likely serving instead as a MAR/PAR reader and potentially as a transcriptional co-regulator in plants. To explore the conservation of RCD1′s mechanism of action, this study assessed its PAR binding capability and PARP activity. Consistent with recent findings, both GmRCD1 and AtRCD1 are capable of binding PAR but do not exhibit PARP activity, suggesting that RCD1 may contribute to regulatory processes through mechanisms other than PARP activity, possibly functioning as a reader protein [60]. Based on the conserved nature of the RCD1 protein’s RST domain, the consistency of its interactions across different species, and its lack of PARP activity, it can be inferred that the function of RCD1 is relatively conserved. However, the current research is confined to in vitro experiments; further in vivo studies are required to validate these findings and to determine which specific domain is responsible for PAR binding.

## 4. Materials and Methods

All primers used in this study are listed in Appendix A. The vectors pET28a, pGEX4T-1, pGBKT7, pGADT7, pGBKT7-p53, pGBKT7-lam, pGADT7-T, pCAMBIA2300-eGFP, and PVYNE/PVYCE were provided by our laboratory.

The plant materials utilized in this study encompassed “*Bainong No. 6*” soybean, *Nicotiana benthamiana*, *Arabidopsis thaliana* (*Columbia-0*), and the Arabidopsis mutant *rcd1-3* (*SALK_116432*), all of which were sourced from our laboratory.

### 4.1. Plant Growth Conditions

Seeds of *Arabidopsis thaliana* were surface-sterilized using a solution of ethanol and sodium hypochlorite, followed by multiple rinses with sterile distilled water. The sterilized seeds were then sown on half-strength Murashige and Skoog (1/2 MS) medium and subjected to vernalization in the dark at 4 °C for 48 h. Following vernalization, the plates were transferred to a controlled environment chamber maintained at 22 °C with 60–70% relative humidity and a photoperiod of 15 h light and 9 h dark for a 10-day growth period. After the initial growth period, the seedlings were transplanted into a 1:1 (*v*/*v*) mixture of peat soil and vermiculite for continued cultivation. Seeds of *Nicotiana benthamiana* were directly sown into the same peat soil and vermiculite mixture and grown under the same environmental conditions as described for Arabidopsis.

### 4.2. Yeast Two-Hybrid Assay

The GmRCD1 gene was amplified via PCR using primers BDG-mRCD1F and BDG-mRCD1R from a soybean cDNA library derived from 15-day-old seedlings subjected to high-salt stress (150 mM NaCl). The PCR product was purified and subsequently cloned into the pEASY-Blunt vector, followed by transformation into Escherichia coli DH5α. Positive clones were identified through colony PCR using primers M13F and M13R and verified by DNA sequencing. The GmRCD1 gene was subcloned into the bait vector pGBKT7, and the construct was confirmed by sequencing. Competent cells of the yeast strain Y2H GOLD were prepared using the Clontech yeast competent cell preparation kit and transformed with pGBKT7-GmRCD1 to assess self-activation on selective media lacking adenine, histidine, leucine, and tryptophan, supplemented with Aureobasidin A and X-α-Gal. For the yeast two-hybrid screening, pGBKT7-GmRCD1 and the soybean cDNA library were co-transformed into Y2H GOLD yeast cells. Interacting clones were selected on quadruple dropout media (-Ade/-His/-Leu/-Trp) containing X-α-Gal, where positive interactions were indicated by the formation of blue colonies, and these clones were subsequently sequenced. To validate the interactions, the target genes from the positive clones were amplified via PCR, cloned into the prey vector pGADT7, and co-transformed with pGBKT7-GmRCD1 into Y2H GOLD yeast cells. Positive interactions were confirmed by the ability of the co-transformants to grow and produce blue colonies on the selective media.

### 4.3. Subcellular Localization Analysis

The target genes GmRCD1 and GmNAC058 were amplified by PCR from the plasmids pGBKT7-GmRCD1 and pGADT7-GmNAC058 and cloned into the pCAM-BIA2300-eGFP vector to create GFP fusion constructs. The recombinant plasmids were introduced into Agrobacterium tumefaciens strain GV3101, and positive transformants were selected and preserved. The Agrobacterium strains harboring the constructs were cultured, harvested by centrifugation, and resuspended in MMA buffer (10 mM MES, 10 mM MgCl_2_, 100 μM acetosyringone, pH 5.6). The bacterial suspension was infiltrated into the leaves of 4-week-old Nicotiana benthamiana plants using a needleless syringe. The infiltrated plants were then maintained at 22 °C under 60% relative humidity with a 16-h light/8-h dark photoperiod for 3 to 5 days. The subcellular localization of the GFP-tagged proteins was examined using a confocal laser scanning microscope (Zeiss LSM 710 Confocal Microscope, Jena, Germany, excitation at 488 nm, emission at 500–550 nm). An empty pCAMBIA2300-eGFP vector was used as a negative control. The acquired images were analyzed using ImageJ software (v1.52p) to determine the subcellular localization of the fusion proteins.

### 4.4. His-Tagged Proteins Induction and Purification

The GmRCD1 gene was amplified via polymerase chain reaction (PCR) from the pGBKT7-GmRCD1 plasmid and subsequently cloned into the pET28a vector to generate the recombinant plasmid pET28a-GmRCD1. Following sequence confirmation, the recombinant plasmid was transformed into Escherichia coli BL21 (DE3) competent cells. Protein expression was induced by the addition of isopropyl β-D-1-thiogalactopyranoside (IPTG) in Luria-Bertani (LB) medium supplemented with kanamycin, and successful expression was verified through sodium dodecyl sulfate–polyacrylamide gel electrophoresis (SDS-PAGE). For purification, the induced cells were lysed via sonication in a lysis buffer (50 mM Tris-HCl, 300 mM NaCl, 10 mM imidazole, pH 8.0), and the clarified supernatant was applied to a nickel-nitrilotriacetic acid (Ni-NTA) affinity chromatography column. The target protein was eluted with a buffer containing imidazole, subsequently exchanged into phosphate-buffered saline (PBS) via ultrafiltration, and stored at −80 °C.

### 4.5. GST-Tagged Proteins Induction and Purification

The GmNAC058 gene was amplified via PCR from the pGADT7-GmNAC058 plasmid using gene-specific primers and subsequently ligated into the pGEX4T-1 vector to create a recombinant plasmid encoding a glutathione S-transferase (GST)-fusion protein. Following sequence confirmation, the recombinant plasmid was transformed into Escherichia coli TransB competent cells. Expression of the GST-fusion protein was induced with IPTG in LB medium supplemented with ampicillin, and successful expression was confirmed via SDS-PAGE. For purification, the induced cells were lysed via sonication in phosphate-buffered saline (PBS) containing dithiothreitol (DTT) at pH 7.4, and the filtered supernatant was applied to a GST affinity chromatography column. The protein was eluted with reduced glutathione (10 mM reduced glutathione, 50 mM Tris-HCl, pH 8.0), subsequently exchanged into PBS via ultrafiltration, and stored at −80 °C. Additionally, the Escherichia coli TransB strain harboring the empty pGEX4T-1 vector was induced under identical conditions, and the GST-tag protein was purified using the same procedure.

### 4.6. GST Pull-Down

Glutathione agarose beads were employed to capture the GST-GmNAC058 fusion protein or the GST empty vector (negative control) in PBS buffer supplemented with 1% Triton X-100. After washing the beads with PBS to remove unbound proteins, they were incubated with a crude extract containing HIS-GmRCD1, followed by additional washes to minimize non-specific interactions. The proteins bound to the beads were eluted using SDS-PAGE sample buffer, resolved on a 12% SDS-PAGE gel, and subsequently analyzed through Coomassie blue staining and Western blotting. In the Western blot analysis, proteins were transferred to a PVDF membrane, blocked with 5% non-fat milk in TBST, and probed with primary antibodies against His-tag (mouse) and GST-tag (rabbit), followed by incubation with HRP-conjugated secondary antibodies. The membrane was then incubated with ECL Plus substrate, and chemiluminescent signals were captured using a pre-cooled CCD imaging system (−30 °C), with band intensities quantified using Image Lab 6.0 software.

### 4.7. BiFC

The genes were amplified by PCR from pGBKT7-GmRCD1 and pGADT7-GmNAC058 and ligated into the binary vectors PVYCE and PVYNE to construct the recombinant plasmids PVYCE-GmRCD1 and PVYNE-GmNAC058. These recombinant plasmids were transformed into Escherichia coli DH5α for propagation and sequence verification. Protoplasts were isolated from the fully expanded rosette leaves of 3- to 5-week-old Arabidopsis thaliana (*Col-0*) plants using an enzymatic solution consisting of 1.5% cellulase R10, 0.4% macerozyme R10, 0.4 M mannitol, 20 mM KCl, 20 mM MES (pH 5.7), 10 mM CaCl_2_, and 0.1% BSA, incubated at 22 °C for 2–3 h. The isolated protoplasts were transfected with the BiFC constructs using a PEG-CaCl_2_-mediated transformation method (40% PEG 4000, 0.2 M mannitol, 100 mM CaCl_2_) and subsequently cultured at 22 °C under continuous light for 16 h. Fluorescence complementation signals were observed using a confocal laser scanning microscope (Zeiss LSM 710 Confocal Microscope, Jena, Germany, excitation wavelength 488 nm/561 nm, emission wavelength 500–550 nm/570–620 nm), with co-transfection of empty PVYCE and PVYNE vectors serving as a negative control.

### 4.8. PAR Binding Capacity Assay

After determining the concentration of the purified recombinant proteins His-AtRCD1, His-GmRCD1, and the negative control GST protein (expressed from an empty vector) using a BCA kit (BCA Protein Assay Kit, Sangon Biotech, catalog number C503021-0500, Shanghai, China) 500 ng of each was spotted onto nitrocellulose membranes (0.45 μm pore size), with three parallel membranes set for each sample. After spotting, the proteins were fixed using a UV crosslinker (120 mJ/cm^2^) for 2 h. Then, the membranes were rinsed three times (5 min each) with TBST buffer (10 mM Tris-HCl, pH 7.4, 150 mM NaCl, 0.05% Tween 20) to remove unbound impurities. The membranes were incubated with 100 nM poly ADP-ribose (PAR, Trevigen, catalog number 4336-100-01) at room temperature for 1 h to allow PAR to bind to the target proteins. After incubation, the membranes were washed five times (10 min each) with TBST and then with high-salt TBST containing 1 M NaCl to reduce non-specific binding. After blocking the membranes with TBST containing 5% skim milk powder for 1 h, they were incubated overnight at 4 °C with primary antibodies (mouse anti-PAR antibody 1:1000, mouse anti-His antibody 1:5000, rabbit anti-GST antibody 1:5000). The next day, the membranes were washed three times with TBST (10 min each), incubated with HRP-conjugated secondary antibody (1:10,000) at room temperature for 2 h, developed with ECL chemiluminescence, and the signal intensity was analyzed using Image Lab software. In the experiment, GST protein served as a negative control to exclude non-specific binding; the high-salt washing step significantly reduced background signals, ensuring the specificity of the results.

### 4.9. PARP Activity Assay

The enzymatic activity of His-AtRCD1 and His-GmRCD1 was quantitatively analyzed using the PARP activity assay kit (Colorimetric PARP Assay Kit, Sigma-Aldrich, catalog number TACS-1005). First, using purified His-empty vector protein as a negative control, standards of 0, 0.025, 0.05, 0.075, and 0.1 U/well were prepared according to the kit instructions, and after incubating at room temperature for 30 min, a standard curve was plotted (R^2^ ≥ 0.99). In the experimental group, the activities of purified His-AtRCD1 and His-GmRCD1 proteins were converted to 0.025 U/25 μL and 0.05 U/25 μL, respectively. In a 96-well plate, 50 μL of 1× PARP buffer was added to each well to rehydrate the histone for 30 min. After discarding the buffer, 0.5 U/well of PARP enzyme (positive control) or the test protein was added, followed by 25 μL of 11× PARP reaction solution (containing NAD⁺ and substrate), and incubated at room temperature for 60 min. After the reaction, the wells were washed twice with PBS containing 0.1% Triton X-100 (200 μL/well) and twice with PBS (5 min each) to thoroughly remove unbound substances. Then 50 μL of 1× Strep-HRP solution was added to each well and incubated at room temperature in the dark for 60 min. After washing, 50 μL of TACS colorimetric substrate was added and developed in the dark for 15 min. The reaction was stopped with 50 μL of 0.2 M HCl, and the absorbance was measured at 450 nm. The absorbance value of the negative control group (His-empty vector protein) was used to subtract the background, ensuring the accuracy of enzyme activity calculation.

## 5. Conclusions

This study represents a pioneering investigation into the molecular interactions between soybean (*Glycine max*) proteins GmRCD1 and GmNAC058, providing critical insights into their roles in enhancing plant resistance to abiotic stress. Utilizing yeast two-hybrid screening of a soybean cDNA library derived from plants subjected to salt stress, we identified 17 potential proteins that interact with GmRCD1, among which the NAC transcription factor GmNAC058 was identified as a key interacting partner. The direct interaction between GmRCD1 and GmNAC058 was confirmed through GST pull-down and bimolecular fluorescence complementation (BiFC) assays, which demonstrated that this interaction is facilitated by the RST domain of GmRCD1 and the C-terminal disordered region (amino acids 288–323) of GmNAC058. Subcellular localization studies revealed that both GmRCD1 and GmNAC058 are predominantly localized in the nucleus, suggesting a potential collaborative role in transcriptional regulation. Additionally, we demonstrated that GmRCD1 binds to poly ADP-ribose (PAR) but lacks catalytic PARP activity, suggesting a non-enzymatic regulatory function in plant stress responses. These findings offer valuable insights into the intricate protein interaction networks underlying plant stress resistance and establish a theoretical framework for developing novel strategies to enhance plant tolerance to abiotic stresses. This research holds significant implications for improving soybeans’ tolerance to environmental stresses through molecular breeding approaches, with potential applicability to other agriculturally important crops.

## Figures and Tables

**Table 1:** Proteins Identified by Yeast Two-Hybrid Screening.

No.	Gene ID	Classification
11	Glyma09g34000	GmSRO1
8	Glyma04g39140	NAC transcription factor
25	Glyma08g16630	NAC transcription factor
7,14,21	Glyma01g36410	ubiquitin–proteasome system protein
20	Glyma15g39830	ubiquitin–proteasome system protein
22	Glyma08g08430	ubiquitin–proteasome system protein
27	Glyma05g36490	ubiquitin–proteasome system protein
8,17,24	Glyma17g04190	ribosomal protein
5	Glyma02g01160	SPL1 transcription factor
30	Glyma14g24710	zinc finger-containing protein
29	Glyma04g01380	NmrA domain-containing protein
12	Glyma11g20030	heavy metal transporter protein
16	Glyma01g44680	protein NETWORKED 1A-like
18	Glyma11g00910	protein NETWORKED 1A-like
15	Glyma18g52221	hypothetical protein
2	Glyma07g33050	hypothetical protein
4	Glyma16g11160	hypothetical protein

**Figure A1:**
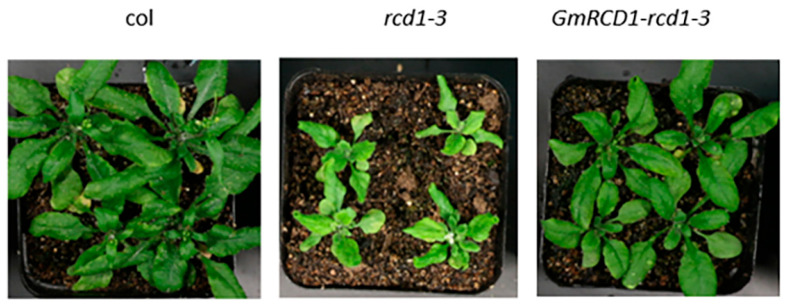
Growth status of different Arabidopsis thaliana. (**left**) Growth of Col wild-type Arabidopsis thaliana. (**middle**) Growth status of Arabidopsis mutant *rcd1-3*. The *rcd1-3* mutant exhibits impaired development compared to wild-type Arabidopsis, characterized by stunted growth, curled leaves, and premature flowering. (**right**) Growth status of *GmRCD1-rcd1-3* supplementation. Overexpression of GmRCD1 in the *rcd1-3* mutant background complemented the mutant phenotype, resulting in plants that phenotypically resembled wild-type Arabidopsis.

## Data Availability

The original contributions presented in this study are included in the article. Further inquiries can be directed to the corresponding author.

## Abbreviations

The following abbreviations are used in this manuscript:

SRO	Similar To RCD One
RCD1	Radical Cell Death 1
NAC	Nascent Polypeptide-associated Complex
MDS	Mitochondrial Dysfunction Stimulon
PARP	Poly ADP-ribose Polymerase
BiFC	Bimolecular Fluorescence Complementation
SPL1	Squamosa Promoter Binding Protein-like 1
ROS	Reactive Oxygen Species
NPR1	Nonexpresser Of PR Genes 1

## Appendix A

**Table A1:** DNA cloning and yeast two-hybrid primer sequences.

Target Gene	Primer Name	Primer Sequence
Glyma09g34000	BDGmSRO1F BDGmSRO1R	TCTAGAATGGAAGCAAAAATCGCAAAGGC GGATCCGACTTCCTTCTTGATTGAGC
Glyma08g16630	GmRY3F GmRY3R	GAATTCATGGTTAAAACTGTTGGAGTTC CTCGAGTCAAGGTTGTGTGGTCGGATA
Glyma01g36410	GmRY4F GmRY4R	GGATCCATGGGAGGTGACGCCACTGCCG CTCGAGCTACTGTCCGGAGTTCCCCAAA
Glyma15g38830	GmRY5NF GmRY5NR	GGATCCATGGGTAATAAACTTGGGAGG GAATTCCTACTGCCAAGAAGCACTCTG
Glyma08g08430	GmRY6F GmRY6R	GGATCCATGGTGAAAATCTCACTTGGTGGA GTCGACTTAATGGTGTGACTTTATGCGAAT
Glyma05g36490	GmRY7NF GmRY7NR	GAATTCATGAGTTCGTCTTCGGCAGATGTTTTTCC CTCGAGCTACTGAGATTCATCCATTGGGTCA
GmNAC82(154–323)	GmNAC82(154)F GmNAC82R	GATACATATGGAAGGTCTTGGTCCCGGG GATAGGATCCTCAAGGTTGTGTGGTCGGATA
GmNAC82(1–287)	GmNAC82F GmNAC82(287)R	GATACATATGGTTAAAACTGTTGGAGTTC GATAGGATCCAGCAAAGTAGTCTTCCAAGCCC
GmRCD1	BDGmRCD1F BDGmRCD1R	TCTAGAATGGAAGCAAAAATCGCAAAGGC GGATCCGACTTCCTTCTTGATTGAGC
GmRCD1W(70–160)	BDGmRCD1WF BDGmRCD1WR	CCATGGGACGGTCCTTAGCTAGGCGT GGATCCTCAGGGGAAAAAACAGCACCCTGC
GmRCD1P(260–460)	BDGmRCD1PF BDGmRCD1PR	CCATGGATGCTTATACTGAATCAGT GGATCCTCAACTTCCACAAAAATGACCTT
GmRCD1R(491–583)	BDGmRCD1RF BDGmRCD1RR	CCATGGCTCCTAGTATGGTTGCTAGCAC GGATCCTCAGACTTCCTTCTTGATTGAGC

**Table A2:** Subcellular localization primer sequences.

Target Gene	Primer Name	Primer Sequence
GmRCD1	GmRCD1GF GmRCD1GR	GGATCCATGGAAGCAAAAATCGCAAAGGC CTCGAGTCAGTCTTCTTTCTTGATTGAGTCTT
GmNAC82	GmNAC82-XbaI-F GmNAC82-BamHI-R	TCTAGAATGGTTAAAACTGTTGGAGTTC GGATCCAGGTTGTGTGGTCGGATA

**Table A3:** Protein expression primer sequences.

Target Gene	Primer Name	Primer Sequence
GmRCD1	GmRCD1GF GmRCD1HR	GGATCCATGGAAGCAAAAATCGCAAAGGC GAGCTCTCAGACTTCCTTCTTGATTGAGC
GmNAC82	GSTGmNAC82F GSTGmNAC82R	GAATTCATGGTTAAAACTGTTGGAGTTC CTCGAGTCAAGGTTGTGTGGTCGGATA

**Table A4:** Bimolecular fluorescence complementation primer sequences.

Target Gene	Primer Name	Primer Sequence
GmRCD1	GmRCD1PF GmRCD1PR	GGATCCATGGAAGCAAAAATCGCAAAGGC CTCGAGGACTTCCTTCTTGATTGAGC
GmNAC82	GmNAC82PF GmNAC82PR	TCTAGAATGGTTAAAACTGTTGGAGTTC GGATCCAGGTTGTGTGGTCGGATA
